# Timely completion of multiple life-saving interventions for traumatic haemorrhagic shock: a retrospective cohort study

**DOI:** 10.1186/s41038-019-0160-5

**Published:** 2019-07-18

**Authors:** Biswadev Mitra, Jordan Bade-Boon, Mark C. Fitzgerald, Ben Beck, Peter A. Cameron

**Affiliations:** 1 National Trauma Research Institute, The Alfred Hospital, 89 Commercial Road, Melbourne, VIC 3004 Australia; 2 Emergency & Trauma Centre, The Alfred Hospital, Melbourne, Australia; 3 School of Public Health and Preventive Medicine, Monash University, Melbourne, Australia; 4 Trauma Service, The Alfred Hospital, Melbourne, Australia; 5 Faculty of Medicine, Laval University, Quebec City, Quebec Canada

**Keywords:** Wounds, Bundle of care, Haemorrhage shock, Resuscitation, Emergency department, Blood products, Timely life-saving interventions, Trauma, Injuries

## Abstract

**Background:**

Early control of haemorrhage and optimisation of physiology are guiding principles of resuscitation after injury. Improved outcomes have been previously associated with single, timely interventions. The aim of this study was to assess the association between multiple timely life-saving interventions (LSIs) and outcomes of traumatic haemorrhagic shock patients.

**Methods:**

A retrospective cohort study was undertaken of injured patients with haemorrhagic shock who presented to Alfered Emergency & Trauma Centre between July 01, 2010 and July 31, 2014. LSIs studied included chest decompression, control of external haemorrhage, pelvic binder application, transfusion of red cells and coagulation products and surgical control of bleeding through angio-embolisation or operative intervention. The primary exposure variable was timely initiation of ≥ 50% of the indicated interventions. The association between the primary exposure variable and outcome of death at hospital discharge was adjusted for potential confounders using multivariable logistic regression analysis. The association between total pre-hospital times and pre-hospital care times (time from ambulance at scene to trauma centre), in-hospital mortality and timely initiation of ≥ 50% of the indicated interventions were assessed.

**Results:**

Of the 168 patients, 54 (32.1%) patients had ≥ 50% of indicated LSI completed within the specified time period. Timely delivery of LSI was independently associated with improved survival to hospital discharge (adjusted odds ratio (OR) for in-hospital death 0.17; 95% confidence interval (CI) 0.03–0.83; *p* = 0.028). This association was independent of patient age, pre-hospital care time, injury severity score, initial serum lactate levels and coagulopathy. Among patients with pre-hospital time of ≥ 2 h, 2 (3.6%) received timely LSIs. Pre-hospital care times of ≥ 2 h were associated with delayed LSIs and with in-hospital death (unadjusted OR 4.3; 95% CI 1.4–13.0).

**Conclusions:**

Timely completion of LSI when indicated was completed in a small proportion of patients and reflects previous research demonstrating delayed processes and errors even in advanced trauma systems. Timely delivery of a high proportion of LSIs was associated with improved outcomes among patients presenting with haemorrhagic shock after injury. Provision of LSIs in the pre-hospital phase of trauma care has the potential to improve outcomes.

## Background

Haemorrhage is responsible for up to 50% of deaths after injury, and of these deaths, about half occur during the early stages of resuscitation [1]. Among those who reach the hospital, early mortality has been associated with continued haemorrhage, coagulopathy and incomplete resuscitation [2]. Once the injured patient develops the ‘triad of death’, outcomes are significantly worse and reversal of coagulopathy and control of haemorrhage becomes exceedingly difficult and, in some cases, efforts may be futile [3, 4].

Early control of haemorrhage and optimisation of physiology are therefore guiding principles of trauma resuscitation. Following airway maintenance and cervical spine control, chest decompression is critical when indicated [5]. The early maintenance of circulation and haemorrhage control involves control of external haemorrhage and anatomical approximation of fractures associated with bleeding, including splinting of the pelvis. These initial steps are able to be performed pre-hospital by most advanced emergency medical services.

In addition, the replacement of circulatory volume to maintain physiologically appropriate perfusion is recommended. Current evidence discourages the infusion of large volumes of crystalloid solutions with emphasis on early transfusion of red cells and pre-emptive transfusion of coagulation products to prevent and treat coagulopathy [6]. Red cell concentrates are available in some pre-hospital services, while availability of coagulation factors is uncommon. The ultimate aim of such interventions is to optimise physiology until definitive control of bleeding, with angiography or damage control surgery required in a small proportion of patients.

In contrast to single interventions, bundles of care for resuscitation after haemorrhagic shock and severe brain injury have been previously proposed [7]. Such bundles outline treatment recommendations when a single pathological process such as haemorrhagic shock or traumatic brain injury has been identified. Improved outcomes have been associated with compliance with such bundles [8]. At the same time, shorter times to isolated life-saving interventions (LSIs) have been associated with improved outcomes [9–13]. However, during the initial period of trauma resuscitation, either in the pre-hospital phase or during reception in the emergency department (ED), multiple LSIs are required for treatment of patients with injuries to multiple body regions [14]. Delays to completion of multiple critical interventions for such complex patients may be substantial and associated with poor outcomes [15–17]. In the absence of large trauma teams in well-resourced trauma centres, such interventions may not be able to be performed simultaneously but prioritised to be performed as soon as possible.

Even in advanced trauma systems, preventable mortality has been associated with failure to successfully intubate, secure or protect an airway, delayed operative or angiographic control of acute abdominal/pelvic haemorrhage and delayed intervention for ongoing intrathoracic haemorrhage [18]. However, in contrast to single interventions, the effect on mortality of multiple LSIs in a specified time period has not been adequately assessed. This study aimed to analyse patients with traumatic haemorrhagic shock and the association between timely initiation of defined LSIs and mortality.

## Methods

### Setting

This was a retrospective cohort study conducted within the Victoria State Trauma System that delivers more than 80% of severely injured patients to two adult major trauma services. Ambulance Victoria triages and transports all suspected adult major trauma patients directly to an adult Major Trauma Service when the travel time is less than 45 min. The Alfred Emergency & Trauma Centre (E&TC) admits in excess of 7500 trauma patients per year with over 1300 patients having an injury severity score of > 12 (abbreviated injury scale (AIS) 2008) [19]. The Alfred Hospital Trauma Registry (AHTR) staff collect trauma data concurrent with the inpatient episode. Regularly audited data are prospectively collected according to a defined dataset by experienced registry staff. AHTR staff collect data on all patients admitted for more than 24 h to The Alfred Intensive Care Unit or trauma patients with an Injury Severity Score (ISS) of more than 12 (AIS 2008) or trauma patients who die during the admission or all trauma patients requiring life-saving operative intervention. All severely injured patients immediately undergo a pre-defined set of pathology tests upon arrival to the E&TC [20]. This study was reviewed and approved by the Alfred Hospital Research & Ethics Committee.

### Inclusion and exclusion criteria

All patients presenting to the E&TC directly from the scene of injury between July 1, 2010 and July 31, 2014 and entered into the AHTR were included. The population was limited to adult patients with haemorrhagic shock by including only patients with an initial (pre-hospital) systolic blood pressure (SBP) of < 100 mmHg and a heart rate (HR) of ≥ 100 beats/min. This definition of haemorrhagic shock based on a combination of hypotension and shock index has been previously proposed and shown to correlate with transfusion requirement in this population [21–24]. Patients with a diagnosed severe head injury (AIS of 5 or 6) were excluded from analysis. Also excluded were patients satisfying the above criteria, but with ISS < 12 and not undergoing any of the specified LSIs in the first 24 h [25].

### Exposure variable

The exposure variable was defined as timely initiation of ≥ 50% of indicated LSIs. An intervention was considered to be indicated if performed within the first 12 h post injury. Time point 0 was taken as the best estimated time of injury as documented in pre-hospital clinical records, and the timing of each intervention when first started, either pre-hospital or in-hospital, was recorded. The LSIs and time limits are listed in Table 1. The list was selected from the key management protocols for breathing, ventilation and circulation in the Advanced Trauma Life Support Student Course Manual (8th edition), American College of Surgeons 2008 [26]. Pre-hospital time was defined as estimated time of injury to arrival time at the trauma centre. Pre-hospital care time was defined as arrival of paramedics at the scene to arrival at the trauma centre.

**Table 1:** Critical interventions for haemorrhagic shock

Intervention	Time limit from estimated injury time
Chest decompression	Within 60 min
External haemorrhage control and/or pelvic splint	Within 60 min
Red cell transfusion	Within 120 min
Coagulation product transfusion	Within 120 min
Angiography and embolization or operating room	Within 180 min

Indications for each intervention were dictated by local guidelines. Chest decompression was performed using set indications by pre-hospital clinical staff using needle thoracostomy and in-hospital after blunt dissection and digital decompression [27, 28]. Ambulance Victoria guidelines recommend application of a pelvic binder if a pelvic fracture is suspected. Resuscitation with red cells, in conjunction with a high ratio of plasma, is recommended in all massive haemorrhage protocols, with indications being poor response to initial crystalloid resuscitation [29]. Red cells were available in pre-hospital aeromedical services during the study period, but not in pre-hospital ground transport vehicles. Following early control of bleeding, restrictive volume replacement and prevention or early management of coagulopathy operative control of bleeding with adjunct use of interventional radiology were considered optimal practice [30].

Given the absence of reliable evidence for appropriate time limits to perform these interventions, conservative limits were set a priori by the authors of this study. This group of LSIs focused on critical treatment only and not investigations. Chest decompression was recorded as being initiated for any needle, finger or tube thoracostomy. External haemorrhage control was recorded if any external pressure or tourniquet was applied, Pelvic binder placement was defined as specialised device or sheet application was documented. Coagulation products included fresh frozen plasma (FFP), platelets and cryoprecipitate. As this trauma centre is currently enrolling in the pre-hospital anti-fibrinolytics for traumatic coagulopathy & haemorrhage (PATCH)-Trauma trial, E&TC administration of tranexamic acid was not routine and never in the initial stages of resuscitation during the period of this study [31]. Patients in whom < 50% of indicated LSIs were initiated within the defined time frames formed the delayed or comparator group.

### Outcome variables

The primary outcome variable was in-hospital death. Secondary outcome variables were time to death and length of hospital stay among survivors. Potential confounders are listed. This list was kept parsimonious to account for the expected small sample of patients with the outcome variable of interest. ISS were based on AIS 2005: update 2008 and categorised [32]. Initial serum lactate level was used as a measure of acidaemia. Coagulopathy was recorded if the first measured international normalised ratio (INR) was > 1.5. This definition was based on previous studies that had concluded that an elevation of INR > 1.5 (and not mild elevations of > 1.2) was associated with mortality and morbidity after severe trauma [33].

### Statistical analysis

Normally distributed continuous variables were summarised using mean (standard deviation (SD)) and differences between means assessed using Student’s *t* test. Skewed or ordinal variables were summarised using median (interquartile range) and statistical significance assessed using Wilcoxon rank-sum test. Differences in proportions were assessed using the chi-squared test. If potential confounders exhibited a statistical association with the outcome variable (*p* < 0.10) and were not plausibly in the causal pathway between the exposure and outcome variables, they were entered into a multivariable logistic regression model to determine independent associations with the primary outcome variable. Variables potentially in the causative pathway, i.e. pre-hospital times and time to LSIs, were not entered into the regression model but assessed with univariate associations. All analyses were performed using Stata v 11.3 (Statacorp, College Station, TX, USA). A *p* value of < 0.05 was considered to be statistically significant.

## Results

**Fig. 1:**
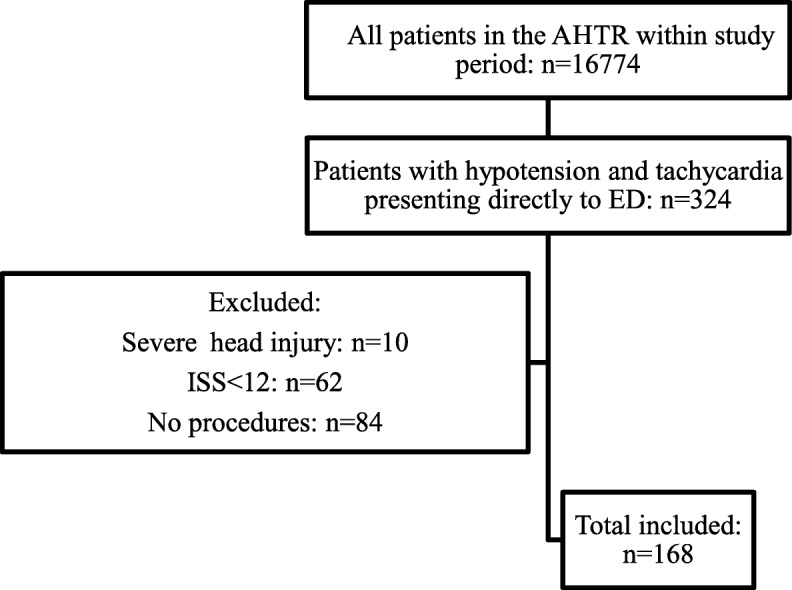
Inclusion of patients for analysis of the association between multiple life-saving interventions and mortality at hospital discharge between July 1, 2010 to July 31, 2014. *AHTR* Alfred Hosptital Trauma Registry, *ED* emergency department, *ISS* injury severity score

Patients eligible for inclusion and exclusion are outlined in Fig. 1. There were 168 patients included in this study. All patients underwent at least one of the pre-defined LSIs as outlined in Table 1. The number of patients that underwent LSIs and the univariate association with in-hospital mortality, together with proportion of timely LSIs, are listed in Table 2. There were 66 patients transported from the scene by helicopter, with no difference in proportion among the exposure and comparator groups. Focussed assessments with sonography for trauma (FAST) scans were performed on all patients on arrival to the E&TC.

**Table 2:** Patients undergoing timely individual interventions and the univariate association with in-hospital mortality

Intervention	Indicated (*n*)	Association with in-hospital mortality (odds ratio; 95% CI)	Completed within time limit, *n* (%)
Chest decompression (60 min)	67	3.89 (1.80–8.38)	30 (44.8%)
External haemorrhage control and/or pelvic splint (60 min)	55	2.57 (1.20–5.47)	33 (60.0%)
Red cell transfusion (120 min)	128	2.32 (0.894–6.44)	50 (39.1%)
Coagulation product transfusion (120 min)	102	1.62 (0.74–3.57)	21 (20.6%)
Angiography and embolization or operating room (180 min)	141	0.07 (0.03–0.19)	30 (21.3%)

*CI* confidence interval

There were 54 (32.1%; 95% confidence interval (CI) 25.5–39.5) patients who received timely LSIs (at least 50% of indicated components performed within the pre-specified time limits). A comparison of patients who had timely interventions compared to those that had delayed interventions is listed in Table 3. Longer total pre-hospital times were associated with delayed LSI (Table 3; *p* < 0.01) and also in-hospital death (unadjusted odds ratio (OR) 1.37; 95% CI 1.02–1.83). Univariable associations between potential covariates to determine the independent association between timely LSI and in-hospital mortality are listed in Table 4. There was no univariate association between timely LSIs and in-hospital mortality (*p* = 0.86).

**Table 3:** Demographic, vital signs and management of patients that underwent life-saving interventions

	Timely life-saving interventions (*n* = 54)	Delayed life-saving interventions (*n* = 114)	*P* value
Age (years)	40.2 (SD 15.7)	40.1 (SD 19.6)	0.97
Male, *n* (%)	45 (83.3%)	76 (66.7%)	0.02
Pre-hospital GCS	13 (IQR 4–14)	13 (IQR 8–14)	0.55
Pre-hospital SBP (mmHg)	63.5 (SD 33.8)	74.0 (SD 25.4)	0.12
Pre-hospital HR (b/min)	122.3 (SD 16.2)	121.9 (SD 16.3)	0.88
Trauma centre SBP (mmHg)	116.4 (SD 48.5)	111.7 (SD 38.5)	0.50
Trauma centre HR (b/min)	107.4 (SD 31.4)	110.4 (SD 29.1)	0.54
Initial lactate (mmol/l)*			0.42
0–2.0	7	24	
2.1–4.0	18	34	
≥ 4.0	22	40	
Coagulopathy (INR > 1.5), *n* (%)	30 (55.5%)	60 (52.6%)	0.72
Initial haemoglobin (g/dl)	113.5 (SD 22.1)	117.7 (SD 27.1)	0.34
ISS			0.17
< 26	12 (22.2%)	42 (36.8%)	
26–35	17 (31.5%)	31 (27.2%)	
36–45	10 (18.5%)	22 (19.3%)	
> 45	15 (27.8%)	19 (16.7%)	
Number of interventions			0.94
1	8 (14.8%)	22 (19.3%)	
2	9 (16.7%)	19 (16.7%)	
3	15 (27.8%)	33 (28.9%)	
4	17 (31.5%)	30 (26.3%)	
5	5 (9.6%)	10 (8.8%)	
Pre-hospital time			< 0.01
< 1.0 h	10 (18.5%)	15 (13.2%)	
1.0 to < 1.5 h	24 (44.4%)	26 (22.8%)	
1.5 to < 2.0 h	18 (33.3%)	28 (24.6%)	
2.0 to < 2.5 h	2 (3.7%)	18 (15.8%)	
≥ 2.5 h	0	27 (23.7%)	
Pre-hospital care time			< 0.01
< 1.0 h	36	38	
1.0–1.5 h	17	39	
1.5–2.0 h	1	17	
≥ 2.0 h	0	20	

*Missing data in 23 patients. Data presented by median ± IQR or mean ± SD

*SD* standard deviation, *IQR* interquartile range, *GCS* glasgow coma scale, *SBP* systolic blood pressure, *HR* heart rate, *INR* international normalised ratio, *ISS *injury severity score

**Table 4:** Association of demographic, vital signs and management variables with in-hospital mortality (univariable analysis)

	Death at hospital discharge (*n* = 36)	Not dead (*n* = 132)	*P* value
Timely life-saving interventions (≥ 50%), *n* (%)	12 (33.3%)	42 (31.8%)	0.86
Age (years)	52.2 (SD 23.4)	36.8 (SD 15.2)	< 0.01
Male, *n* (%)	25 (69.4%)	96 (72.7%)	0.70
Pre-hospital care time (h)	1.3 (SD 0.7)	1.1 (SD 0.5)	0.06
Pre-hospital GCS	6 (IQR 3–14)	13 (IQR 10–14)	< 0.01
Pre-hospital SBP (mmHg)	72.3 (SD 27.0)	64.7 (SD 34)	0.16
Pre-hospital HR (b/min)	121.8 (SD 16.4)	122.7 (SD 15.8)	0.77
Trauma centre SBP (mmHg)	86.9 (SD 62.0)	120.4 (SD 31.1)	< 0.01
Trauma centre HR (b/min)	92.3 (SD 48.1)	114.1 (SD 20.2)	< 0.01
Initial lactate (mmol/l)			< 0.01
0–2.0	4	27	
2.1–4.0	3	49	
≥ 4.0	22	40	
Coagulopathy (INR > 1.5), *n* (%)	30 (83.3%)	60 (45.4%)	< 0.01
Initial haemoglobin (g/dl)	98.8 (SD 30.9)	120.1 (SD 22.8)	< 0.01
ISS			0.06
< 25	9	45	
26–35	6	42	
36–45	9	23	
> 45	12	22	
Number of interventions			0.08
1	3	27	
2	6	22	
3	11	37	
4	9	38	
5	7	8	

*SD* standard deviation, *IQR* interquartile range, *GCS* glasgow coma scale, *SBP* systolic blood pressure, *HR* heart rate, *INR* international normalised ratio, *ISS* injury severity score

Data presented by median ± IQR or mean ± SD

**Fig. 2:**
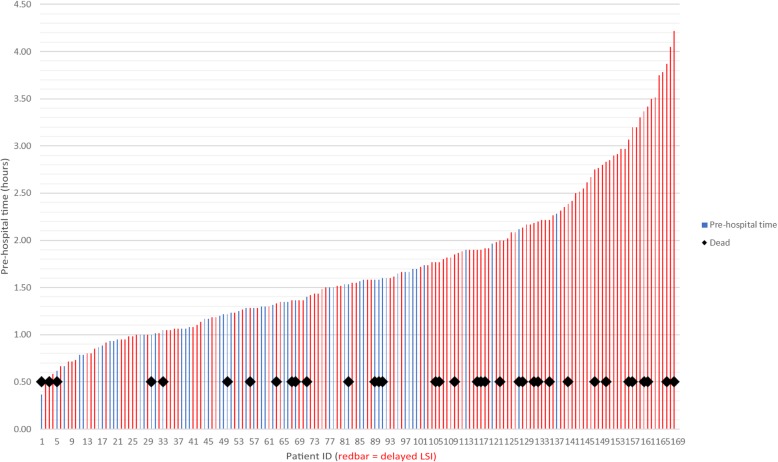
Association between pre-hospital time, delayed life-saving intervention (LSI) and in-hospital death of patients with haemorrhagic shock

There were 36 (21.4%) in-hospital deaths. After adjusting for confounders (age, pre-hospital care time, pre-hospital glasgow coma scale (GCS), initial lactate, ISS and coagulopathy), timely LSIs were associated with reduced odds of death at hospital discharge (OR 0.17; 95% CI 0.03–0.83; *p* = 0.028; Table 5). The Hosmer-Lemeshow test showed a Pearson chi-square of 3.23 and a *p* value of 0.92, indicating good calibration of the model. The association between pre-hospital times, completion of LSIs and in-hospital mortality is illustrated in Fig. 2. Among survivors, hospital length of stay for receiving a high proportion of timely LSIs was 22 (12–33) days and 15 (10–24) days among patients with delayed bundle (*p* = 0.17).

**Table 5:** Adjusted odds ratios for association of variables with in-hospital mortality

Variable	Adjusted odds ratio (95% CI)	*P* value
Timely life-saving interventions	0.17 (0.03–0.83)	0.028
Age	1.09 (1.04–1.13)	< 0.01
Initial GCS	0.82 (0.71–0.94)	0.007
Initial lactate 0–2.0	Reference	
Initial lactate 2.0–4.0	0.11 (0.01–0.94)	0.044
Initial lactate ≥ 4.0	2.29 (0.48–10.89)	0.30
Pre-hospital care time	0.84 (0.29–2.44)	0.75
ISS		
< 25	Reference	
26–35	0.91 (0.13–6.11)	0.92
36–45	2.12 (0.36–12.41)	0.40
> 45	1.36 (0.25–7.29)	0.72
Coagulopathy (INR > 1.2)	5.89 (1.47–23.62)	0.012

*GCS* glasgow coma scale, *INR* international normalised ratio, *ISS* injury severity score, *CI* confidence interval

## Discussion

This study demonstrated a significant association between timely LSIs in severely injured patients with haemorrhagic shock and their subsequent survival. The fact that this is significant, even in a large volume trauma centre, indicates a need for reassessment of the approach to providing care for the most severely injured. This study highlights that major trauma patients may benefit from a heterogeneous set of LSIs, which are needed to be individualised to the patient. Rather than ‘bundles of care’ for all patients, each patient required their ‘own bundle’, with certain components administered, at different times, but a high proportion needing timely delivery to achieve improved outcomes.

In the setting of long pre-hospital times, this research suggests that patients may benefit from LSIs delivered in the pre-hospital phase of trauma care. The findings are consistent with research in the combat setting, where LSIs were deemed to be required by most urgent casualties, and a delay in their performance associated with increased mortality. In this study, delays were noted for airway control, thoracostomy, control of external haemorrhage (tourniquet) and delivery of colloids [34]. The challenges of managing critically ill trauma patients in austere environments were noted.

Civilian guidelines in advanced trauma services recommending that severely injured patients be transported directly to appropriate trauma facilities may therefore subject patients to longer times in resource-poor environments being the back of an ambulance [35]. This not only has been almost universally associated with improved outcomes, but has also resulted in longer pre-hospital times. The combination of time to scene, scene time and then transport time results in long periods of initial care in the pre-hospital phase [36].

While the overarching evidence for primary transport to trauma centres remains strong, in the setting of haemorrhagic shock, strategies need to be individualised depending on time from trauma centres. Injured patients requiring prolonged pre-hospital care are a discrete subset of the military and civilian trauma populations. This may be due to remote location of the patient or delayed discovery after trauma. The pre-hospital phase of resuscitation is critical in these patients. In the military setting, the Special Operations Command Prolonged Field Care Working Group, composed of medical-specialty subject matter experts, has been tasked to evaluate the current training and preparedness of Special Operations Forces (SOF) medics [37]. It is recognised that the capacity to effectively resuscitate injured patients in the setting of PFC requires overarching capabilities and life-saving procedures, including the capability to resuscitate with blood and blood products, ventilate and oxygenate the patient and perform advanced surgical interventions such as tube thoracostomy insertion, fasciotomy, wound debridement and amputations [38].

Critical interventions after major trauma may be delayed due to delayed recognition of the life-threatening pathology, often difficult to detect in the pre-hospital phase of trauma care. Even after arrival to a trauma centre in advanced trauma systems, definitive airway management may be delayed [39]. In some cases, this may be appropriate while the circulation is prioritised, provided the airway is patent [40]. As such, this manuscript focused on LSIs that would be considered mandatory, rather than debatable during resuscitation of patients with haemorrhagic shock. Appropriate administration of blood and blood products is often delayed and associated with poor outcomes [41]. Algorithm based decision support systems may provide a solution, having been proven to reduce errors and improve times to critical interventions and is indicated to be applied to all phases of trauma resuscitation [42]. Such systems have demonstrated improved protocol compliance and reduction of errors of omission. In high-volume trauma centres, continuous presence of specialised staff at all hours improves assessment and management of complex, critically ill patients [43] and, with emerging technology, can readily connect trauma physicians to pre-hospital clinicians for added support [43]. Simple head or chest mounted video could also provide scene support for complex field decision-making. Ongoing improvements in pre-hospital and in-hospital assessments could further improved recognition for the need for LSIs, including point of care tests (such as lactate and viscoelastic measures of coagulation), ultrasound, and perhaps continuous vital signs analysis.

Limitations of this study include its single-centre population and retrospective nature. However, it involves a large proportion of state-wide, severely injured patients. Patients who died at the scene of trauma or in transit were not included in this study. The sample size was limited to patients where haemorrhagic shock would have been obvious from vital signs alone. This was designed to limit bias from delayed recognition resulting in delayed interventions [24]. While we attempted to limit co-variates, the final model was underpowered for the 36 patients in this study with the outcome of interest. Trauma resuscitation involves a complex set of tasks and it is possible that many other LSIs should be considered in some patients, and in its retrospective methodology, this study does not account for unknown confounders.

A distinct population of patients would have required LSIs without meeting our definition of obvious haemorrhagic shock and should form the population for future research. The small number of cases with outcome of interest limited the number of co-variates that could be included into the multivariable logistic regression analysis. The possibilities of significant unknown confounders are therefore high. Additionally, we evaluated timely initiation of care and not the quality of care. While we excluded patients with severe traumatic brain injury defined by AIS (head) 5 and 6, it is possible that head injury of AIS < 5 may have impacted on LSIs. For further assessment of quality, indications of the LSIs, effectiveness of chest decompression, accurate positioning of pelvic binders, blood and blood products in timely ratios and control of haemorrhage through surgery may be more appropriate exposure variables. Early assessments of coagulation disorders and goal-directed haemotherapy algorithms were not in place during the study period but have the potential to further improve patient outcomes [44].

Future research could focus on evidence-based time targets for assessment of and completion of LSIs for patients after traumatic haemorrhagic shock. Although a threshold of 50% was chosen for the proportion of indicated LSIs, in an ideal world, this proportion should be closer to 100%. In the pre-hospital phase, this should not delay scene or transport times, but rather be performed, where possible, en route to hospital. Improvements to current care processes were not specifically assessed in this study including early trauma resuscitation techniques such as open thoracostomies instead of needle decompression, in conjunction with training and audit [45], routine pelvic splints and tourniquets in the presence of shock and the use of haemostatic gauze [46–48]. Consistent evidence highlighting harm from crystalloid resuscitation should translate into alternate fluid therapy in the setting of haemorrhagic shock. Timely management of haemorrhage is widely recommended [49, 50]. The availability of red cells has improved over the last few years with availability in some pre-hospital services but needs to be accompanied by coagulation products, such as freeze-dried plasma and fibrinogen [51–53].

## Conclusions

In a mature trauma system, a small proportion of severely injured patients had LSIs completed within ideal, specified time targets. Timely completion of multiple, rather than single, interventions were associated with improved outcomes. Timely LSIs by pre-hospital staff, in conjunction with primary transport to trauma centres, requires further evaluation in efforts to improve outcomes of severely injured patients in haemorrhagic shock.

## Abbreviations

AHTR: Alfred Hospital Trauma Registry
AIS: Abbreviated Injury Scale
CI: Confidence interval
E&TC: Emergency & Trauma Centre
FAST: Focussed assessments with sonography for trauma
HR: Heart rate
INR: International normalised ratio
ISS: Injury Severity Score
LSI: Life-saving intervention
OR: Odds ratio
SBP: Systolic blood pressure
SOF: Special operations forces
